# Characteristics and Validation Techniques for PCA-Based Gene-Expression Signatures

**DOI:** 10.1155/2017/2354564

**Published:** 2017-02-06

**Authors:** Anders E. Berglund, Eric A. Welsh, Steven A. Eschrich

**Affiliations:** Department of Biostatistics and Bioinformatics, Division of Population Sciences, H. Lee Moffitt Cancer Center and Research Institute, Tampa, FL, USA

## Abstract

*Background*. Many gene-expression signatures exist for describing the biological state of profiled tumors. Principal Component Analysis (PCA) can be used to summarize a gene signature into a single score. Our hypothesis is that gene signatures can be validated when applied to new datasets, using inherent properties of PCA.* Results*. This validation is based on four key concepts. Coherence: elements of a gene signature should be correlated beyond chance. Uniqueness: the general direction of the data being examined can drive most of the observed signal. Robustness: if a gene signature is designed to measure a single biological effect, then this signal should be sufficiently strong and distinct compared to other signals within the signature. Transferability: the derived PCA gene signature score should describe the same biology in the target dataset as it does in the training dataset.* Conclusions*. The proposed validation procedure ensures that PCA-based gene signatures perform as expected when applied to datasets other than those that the signatures were trained upon. Complex signatures, describing multiple independent biological components, are also easily identified.

## 1. Introduction

The use of gene signatures and Principal Component Analysis [1] (PCA) is a popular combination, but a recent publication has clearly shown drawbacks with this combination [2]. Gene signatures are used to represent a biological event and have the potential to describe complex biology better and more robustly than a single gene. There exists a large amount of literature on how to properly analyze microarray data and derive signatures [3–7], validate biomarkers [3, 8, 9], and, in particular, validate prognostic models [10–13], all in response to the poor reproducibility rate in publications [14–18]. A recent report by the Institute of Medicine [19] summarizes many of these issues of signature reproducibility and validation. We will, in this article, focus on how to quantitate the validity of applying PCA-based gene signatures to new datasets. PCA is a technique that reduces a high-dimensional dataset to a low-dimensional dataset while retaining most of the variation in the data. These new variables are referred to as scores, *t*, and the importance (weighting) of the original variables given in the loadings, *p*. For a more in-depth discussion of PCA, we refer to a recent tutorial by Bro and Smilde [20]. PCA models can describe unintended biology when there is large variation due to sources other than the biological process of interest, which can cause random signatures to be significantly associated with outcome [2].

Gene based signatures can be derived using several different techniques. One technique is to include all genes known to be involved in a specific pathway or process, such as a signaling pathway, and treat the signature as a representation of pathway activation. Gene signatures can also be derived from cell line experiments, where a specific biological event is being modulated, or by comparing different known mutation types within the cell lines. In this case, the signature is usually derived from a list of statistically significant differentially expressed genes.

In cancer research, these gene signatures can be used, for example, to predict tumor chemotherapy resistance, aggressiveness, and several other types of clinically relevant scores. These scores are then commonly used for survival analysis, such as Kaplan-Meier plots and log-rank tests. In order to associate these clinical metrics to a gene signature, a single score value is commonly calculated from the individual expression levels of all genes within the signature. One commonly used technique is PCA, where the score vector for the first component is used to represent the gene signature. PCA is a well-established technique for data analysis and has been widely used in many areas. The resulting score can be seen as a weighted average, where each gene is weighted by its importance within the first principal component. There are several advantages to using PCA. For example, not all genes are weighted equally—more important (statistically and, we assume, biologically) genes are weighted higher. It is also robust to noise and can handle both up- and downregulated genes equally well. There are also some pitfalls, due to the inherent properties of PCA, that require special attention. One such pitfall is that if the gene signature is biologically complex, describing more than one biological event, the score from the PCA model may only describe one of the biological events. The PCA model does not necessarily describe all the biological events in the first principal component. Another, probably more well known, issue with PCA models is sign-flipping. The sign of the score value for a sample may change, or flip, depending on the software used and/or small changes in the data. This does not change the interpretation of the PCA model, but it may lead to trouble when comparing different PCA models derived from different datasets and/or software, where the signs may be reversed. The PCA model can easily be flipped back by multiplying both the scores and loadings with −1, a 180-degree rotation.

These issues clearly demonstrate the need to define the ideal characteristics of a PCA-based gene signature and measures of how “well behaved” a signature is when applied to a dataset. Currently, one common way to measure the performance of a signature within cancer tumor datasets is by survival analysis. Even if this is the ultimate goal of a signature, it may be misleading, as was recently shown by Venet et al. [2]. They showed that many random gene-set PCA models were as good as literature-derived and experimentally derived signatures in predicting survival. They ascribed this effect to a proliferation-signature bias present in many tumor datasets. Due to the large number of genes affected by proliferation pathways in tumors, together with large differences in proliferation status between samples, the first principle component of a PCA analysis, PC1, often captures proliferation-related effects in addition to any effects related to the signature of interest. This can result in false-correlation with survival, where the correlation comes more from proliferation-bias effects, rather than from the signature of interest. Due to these issues, it has been shown that gene signatures can be unstable [21] and that single genes can be as good as a multigene signature [22], leading to a recent review on the value of gene-expression signatures by Chibon [14]. This manuscript describes the key aspects of a PCA-based signature, along with a set of measures and figures that will describe how suitable a gene signature is when applied to a given dataset. This includes measures on how robust it is, if the signature is too complex to work well with a PCA model, if the signature differs from the general direction in the dataset, and, most importantly, if the signature describes the same biology that it was intended to. We define a gene signature as a list of genes with corresponding direction and if relevant, magnitude, which are used to describe a biological signal, such as tumor aggressiveness, distant metastasis, survival, or gender.

## 2. Material and Methods

Principal Component Analysis was performed using MATLAB. Gene-expression data consisted of log2 intensities. Mean-centering and unit variance scaling were applied to expression values prior to computing PCA models.

### 2.1. Generation of Randomized Gene Signatures

Results for the gene signature PCA model are compared to thousands of PCA models, based on randomly selected gene signatures. This approach has many benefits, including (1) the performance statistics of PCA, including explained variance, are dependent on many factors and are, thus, not comparable across different datasets and gene signatures. (2) Many of the datasets used are biased in one or several ways, as clearly shown by Venet et al. [2]. Using randomized gene signatures as comparisons removes this bias. (3) It allows for a clear measurement of how much “better” the true gene signature is than randomly generated gene signatures.

Randomized gene signatures were generated by randomly selecting an equal number of genes as the true signature and performing a PCA model for this random gene signature. This is repeated many times to get a distribution of expected values. In this work, we have chosen to use 10,000 randomized gene-sets.

### 2.2. Signatures

We have, in this study, used two signatures, along with some modifications of these signatures, in order to exemplify our results. The two signatures, gender and tumor versus normal, were chosen due to their strong biological signal, common use cases, and potential problems. The tumor versus normal signature largely captures proliferation/cell cycle biology, which is often a dominant feature of tumor biology [2], and gender may be used in quality control analysis, where the gender derived from molecular data is compared to the clinically known gender to identify potential sample mismatches. Each signature was also chosen to highlight a different potential pitfall of PCA analysis: nonuniqueness of signature (tumor versus normal) and absence of meaningful signal when the biology of interest does not vary (gender).

The first signature is a gender signature (Gender-29), consisting of 29 probesets representing 20 unique genes, 19 of which are present in the TCGA RNAseq dataset. To describe the directionality of these genes, we used either −1, for male-specific genes, or 1, for female-specific genes. The second signature was derived from a publically available Tumor/Normal dataset (GSE18842). An all-probeset PCA model of GSE18842 shows a clear separation between tumor and adjacent normal samples in the first principal component (data not shown). The Tumor/Normal (TvsN-100) gene signature was derived by taking the top 100 probesets with the largest absolute loading values for the first principal component. Additionally, we only selected probesets that were also present on the Affymetrix U133A chip. The individual loading value from the first principal component was used to both describe the directionality and the magnitude for each gene. Since proliferation is the major biological process that is different between tumors and adjacent normals, this signature will be related to proliferation. When applied to the TCGA RNAseq dataset, 84 unique genes were used for this signature. The TvsN-100 was also used to generate modified signatures by gradually adding probesets not related to the separation of Tumor/Normal samples. This was done to simulate the addition of random noise to the signature. These are named TvsN-50/50, TvsN-25/75, and TvsN-10/90, respectively, corresponding to the percentages of TvsN-100/unrelated probesets. The last signature is a mix of the Gender-29 and the TvsN-100 signature (Mix-29/29). It was generated by merging all 29 probesets from the Gender-29 signature with the top 29 probesets for the TvsN-100 signature. For this signature, only the directionality was used. To further investigate the effect of mixing two signatures, the number of TvsN-100 probesets was gradually decreased, creating three additional datasets, Mix-29/24, Mix-29/19, and Mix-29/14. These mixed signatures will exemplify a complex signature describing more than one type of biology.

### 2.3. Datasets

Three publically available datasets were used in this study. These were chosen for several reasons: DC (lung tumors, both genders), GSE2034/Breast (same tumor type as data used in Venet et al. [2], single gender), and PRAD (prostate adenocarcinomas, single gender). The rationale for selecting these three datasets is the following: (1) the TvsN-100 signature is expected to perform well on all three datasets, (2) gender signature is expected to perform well on the DC dataset but not on Breast and PRAD datasets, (3) GSE2034/Breast was the same tumor type used by Venet et al. [2], and finally (4) they represent three different tumor types. The first is Director's Challenge (DC) dataset, which consists of 442 lung tumor samples [23] arrayed on the Affymetrix Human Genome U133A Array. This dataset was batch corrected using COMBAT [24], since it shows a clear dependence on the institution where the samples were run. The second dataset is from 286 Breast (Breast) samples (GSE2034) arrayed on the Affymetrix Human Genome U133A Array. Both of these datasets were normalized using IRON [25]. The third dataset is from The Cancer Genome Atlas (TCGA) and contains 297 primary prostate adenocarcinoma (PRAD). The level 3 Illumina HiSeq RNAseqV2 RSEM gene-level normalized mRNA expression data was downloaded from the TCGA data portal in December of 2014.

## 3. Results and Discussion

**Figure 1:**
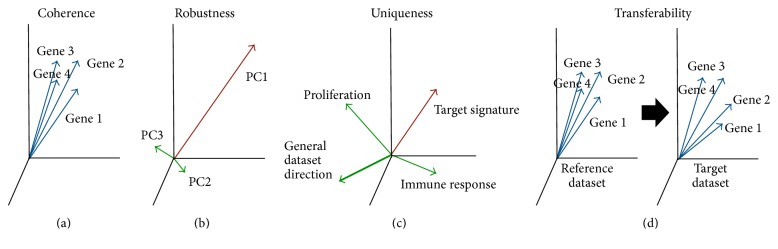
Conceptual view of how a good PCA-based gene signature should behave. The genes in a signature should be coherently expressed within the data (a). A signature should represent a single biological effect (b). A signature should represent unique and distinct signal within the data (c). A signature should represent the same biology as a known reference dataset (d).

As illustrated in Figure 1, we propose several intrinsic characteristics of PCA-based signatures. Rather than focus on correlation of a PCA-based signature with a desired outcome, the characteristics by which a PCA-based signature may be considered valid are examined, independent of endpoint outcome.

### 3.1. Coherence

Individual genes in a gene signature should be correlated beyond chance, as illustrated in Figure 1(a). A coherent gene signature is an indication that a common mechanism or biological pathway is measured.

### 3.2. Robustness

If a gene signature describes more than one distinct biological effect, more than one PC will be significant (Figure 1(b)). PC1 may describe a combination of biological effects, but one effect should predominate. If the explained variances of PC1 and PC2 are similar to each other, this may be an indication that more than one biological effect is present. This is challenging, as the biological effect described in PC1 can easily change (PC1 and PC2 can switch ranking) if a few genes or samples are removed or if the gene signature is applied to a different dataset. Thus, it is preferred for a PCA signature to represent only a single biological effect.

### 3.3. Uniqueness

Datasets may be biased, meaning that many genes are not just random but actually describe a true effect such as proliferation (Venet et al. [2]). This bias can also originate from differences in RNA quality between samples or other types of batch-related effects. It is important to determine if the true gene signature is different from the general direction (Figure 1(c)) in the dataset, in order to determine the uniqueness of the gene signature.

### 3.4. Transferability

It is important that the same biology is present and described by the PCA model as the gene signature was intended to explain (Figure 1(d)). Often, the gene signature is derived from a dataset with controlled variation, for example, a knockout experiment in several different cell lines. In these cases, a reference value can relate (positively or negatively) the biological effect to the gene signature. It is important to define which genes are upregulated versus downregulated, compared to all the genes in the signature.

### 3.5. Measures of PCA-Based Signature Validation

**Figure 2:**
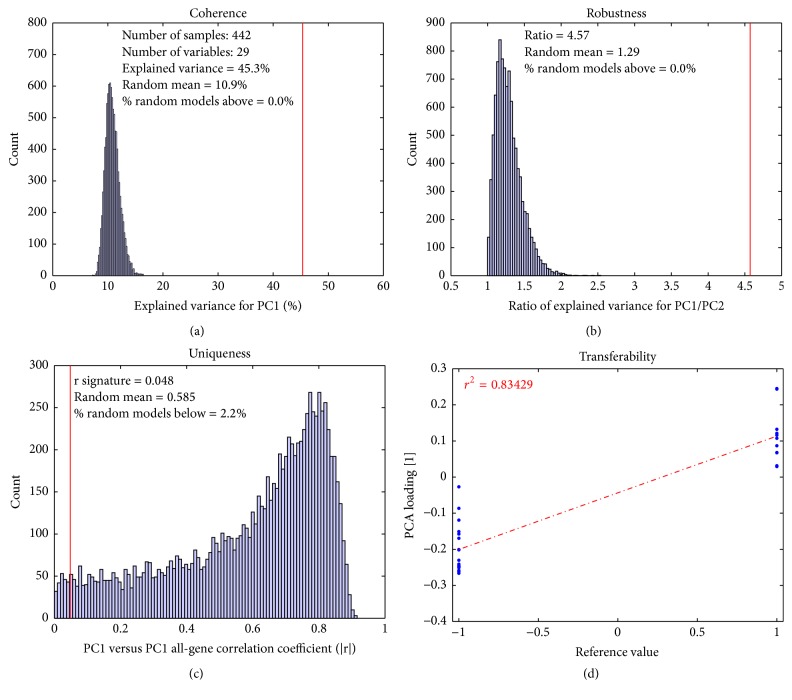
Validation plot for Gender-29 signature applied to DC dataset. The probesets in the Gender-29 signature show a coherent behavior when applied to the DC dataset (a). Furthermore, the PCA model is robust, with a PC1/PC2 ratio of 4.57 (b), and is unique (c). The Gender-29 signature describes gender difference, since the positive (female) probesets are positive in the PCA loadings and the male-specific probesets are negative (d).

Given the 4 characteristics of a PCA-based signature, we developed measures to determine the validity of these characteristics for a given signature when applied to a particular dataset (Table 1). We use the Gender-29 signature as a positive control and example; when applied to a relevant dataset (DC, or lung cancer with mixed gender), it is expected to perform well (Figure 2). Random signatures were used for comparison of PCA model parameters throughout (see Materials and Methods for details). Taken together, the validation results indicate that the Gender-29 signature is a good signature when applied to the DC dataset.

#### 3.5.1. Coherence

For a measure of coherence, the amount of variance explained in the first principal component is used. Increased correlation among variables results in a larger explained variance in the first component, since PCA can be seen as finding the largest eigenvalue to the correlation matrix when the variables are scaled to unit variance [20]. The explained variance for the signature PCA is compared to the distribution of the explained variance of the randomized gene signature PCA models. The coherence figure (Figure 2(a)) shows that the genes in the Gender-29 signature are expressed in a coherent way, since its explained variance is 45.3%. This is larger than any of the randomized gene signature PCA models (mean: 10.9%), and none of the 10,000 random models score higher than the gender signature model in coherence.

#### 3.5.2. Robustness

Our measurement of robustness is simple: the ratio of explained variance between PC1 and PC2. This value should be as large as possible and should also be compared to the distribution of ratios from the randomized gene signature PCA models. The results for the robustness figure (Figure 2(b)) indicate the Gender-29 signature has a PC1/PC2 explained variance ratio of 4.57. This ratio is also higher than that for the randomized gene-set PCA models. This indicates that the gender signature clearly describes a single biological effect in this dataset.

#### 3.5.3. Uniqueness

We use PC1 from a PCA model using all the genes in the dataset as a representation of the overall direction of the dataset. The uniqueness value is derived by calculating the absolute value of the correlation between the true gene signature PCA scores versus the PCA scores of the all-gene model. This is then compared to the distribution of the absolute value correlation between the random gene signature PCA models and the PCA model using all genes. This plot indicates if there is a major variability in the dataset (most random models highly correlated with PCA scores from the all-gene model). This could be a potential problem if this major variability is also correlated to outcome, as was shown by Venet et al. [2].

The uniqueness figure (Figure 2(c)) shows that the Gender-29 signature also differentiates from the general direction of this dataset.

#### 3.5.4. Transferability

The measurement of transferability is the correlation between the PCA loadings for the gene signature versus the reference values. If these are correlated, it implies that the gene signature describes the same biology within the dataset that it was intended to. The PCA loadings are directly related to the importance and directionality of each variable relative to the principal component [20]. This is a direct result from the fact that the scores can be seen as a weighted average of all the variables. The gender signature also describes the same biology (gender) in this dataset, since the transferability figure (Figure 2(d)) shows that the same genes have a positive loading value in both the PCA model and the reference value, and the same is true for the negative values.

In conclusion, the gender signature, when applied to the DC dataset, fulfills all the criteria for being a good signature and describes the correct biology.

### 3.6. The Gender Signature Fails to Translate in the Breast Dataset (Good Signature/Nonrelevant Dataset)

**Figure 3:**
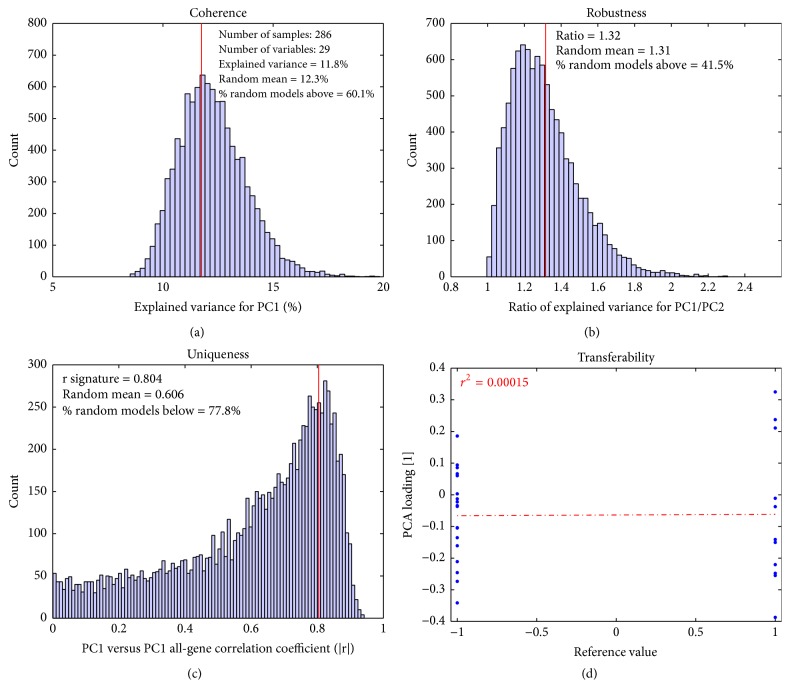
Validation plot for Gender-29 signature applied to Breast dataset. The probesets within the Gender-29 signature are no longer coherent (a), and the ratio of PC1/PC2 is much lower (b). It is also clear that the signature is no longer unique (c). Finally, the biological meaning of the probesets has changed in the Breast dataset (d).

Figure 3 shows the results for the Gender-29 signature when applied to the Breast dataset, which only contains tumor samples from females. It is clear that this signature does not work as intended for the Breast dataset. The* coherence* figure (Figure 3(a)) shows a lower value of 11.8% explained variance for PC1, below the random mean of 12.3%. The* robustness* ratio (Figure 3(b)) is also worse, at 1.32, compared to 4.57 in the DC dataset. More importantly, the* uniqueness* plot (Figure 3(c)) shows that its direction is similar to most of the randomized gene signature PCA models and is correlated to the general direction of the dataset. The* transferability* plot (Figure 3(d)) confirms that this is not a good dataset for the Gender-29 signature, since there is no correlation between the PCA loading and the reference values. All these results indicate that even if the gender signature is a valid signature, it does not work as intended on the Breast dataset. These results also show that random models can be as good as the true model.

### 3.7. Tumor versus Normal Signature Validation in Breast Dataset (Good Signature/Relevant Dataset)

**Figure 4:**
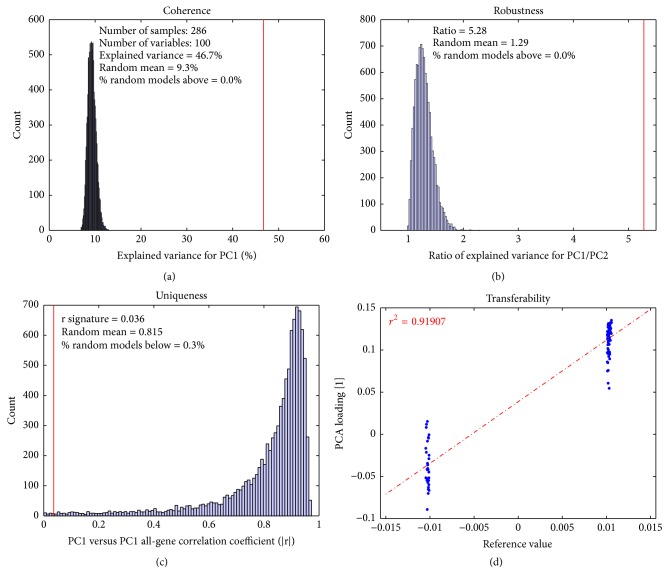
Validation plot for TvsN-100 signature applied to Breast dataset. To demonstrate a signature that is valid within the Breast dataset, the TvsN-100 gene signature was applied. The probesets for the TvsN-100 signature are coherent (a), and the PCA model is robust (b). The signature is also unique (c) and describes the same biology as in the dataset that it was trained upon (d).

To demonstrate that valid gene signatures exist within the Breast dataset, a tumor versus normal (TvsN-100) signature, derived in lung cancer, was applied to the Breast dataset. The results clearly demonstrate that this is a good signature applied to a relevant dataset (Figure 4). The* coherence* is high with values of 46.7%, clearly higher than any of the 10,000 randomized gene-set PCA models. The PCA model is also* robust* with a PC1/PC2 ratio of 5.28, which is higher than any of the random gene signature PCA models. The direction of the TvsN-100 signature is also* unique*, with a correlation coefficient of *r* = 0.036 to the general direction. Finally, the same biology, genes that differentiate tumor samples from normal samples, is also present in this dataset, as shown by the* transferability*. The results are similar to the results seen for the Gender-29 signature when applied to the DC dataset (Figure 2).

### 3.8. Noise Injection Demonstrates the Power of PCA-Based Signatures

To investigate the effect random noise has on a PCA-based signature, we gradually increased the number of nonrelevant probesets in the TvsN-100 signature. The original TvsN-100 signature performed well in the DC dataset, as seen in Table 1 or in Supplementary Figure  1, in Supplementary Material available online at https://doi.org/10.1155/2017/2354564. The new signatures consisted of 50/50, 25/75, and 10/90 original and unrelated probesets. As can be seen in Table 1, both the TvsN-50/50 and the TvsN-25/75 signatures exhibit good statistics. This is also confirmed in the validation plots (Supplementary Fig. S2–S4). It is not until there are only 10 relevant probesets and 90 nonrelevant probesets, TvsN-10/90, that the signature starts to fall apart. This is most visible in the* robustness* measure, with a PC1/PC2 ratio of 1.4, and with 19% of the random PCA models having a better PC1/PC2 ratio. This is further confirmed by calculating the correlation between the original TvsN signature and the modified ones. The correlation to the original TvsN signature is as follows: *r*^2^ = 0.993, *r*^2^ = 0.979, and *r*^2^ = 0.873 for the TvsN-50/50, TvsN-25/75, and TvsN-10/90, respectively (Supplementary Fig. S5). It is also noteworthy to see how the explained variance falls from 58.5% for the original TvsN signature to just 9.4% for the TvsN-10/90 signature. This is expected, since more and more noise is added that is not explained by the first PCA component.

### 3.9. PCA Signatures Do Not Represent Mixed Signatures Robustly (Bad Signature/Relevant Dataset)

**Figure 5:**
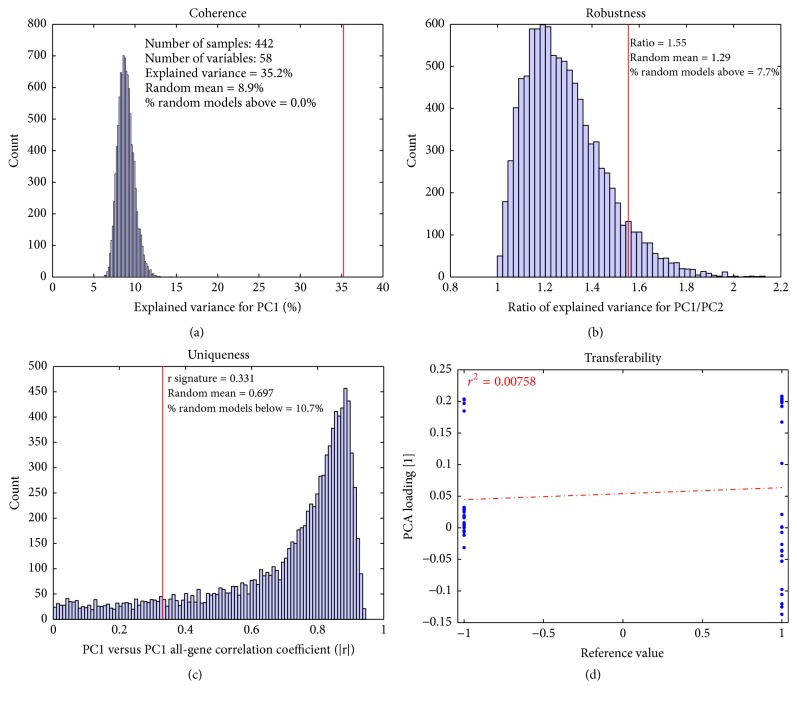
Validation plot for Mix-29/29 signature applied to DC dataset. Merging two “good” signatures and applying the resulting signature to a relevant dataset do not mean that the new signature will be valid. The probesets in the Mix-29/29 signature have a coherent expression (a), but the ratio of PC1/PC2 (b) is lower than that in the individual signatures. The uniqueness is also slightly worse (c). That the Mix-29/29 model is not working as expected is clearly seen in the transferability plot (d), where the PCA model for the DC dataset is not related to reference values.

As was seen in Figure 2 and Supplementary Figure  1, both the Gender-29 and the TvsN-100 signature performed well in the DC dataset. When they are merged into the mixed signature, Mix-29/29, the results are different, as can be seen in Figure 5. The* coherence* is still high, with an explained variance of 35.2%, higher than any of the randomized PCA models. That there are some problems becomes clear in the* robustness* plot (Figure 5(b)), which shows a lower PC1/PC2 ratio (1.55 versus 4.57 and 9.7 for the individual signatures) than before. This is due to the fact that the mixed signature model is not a one-component PCA model, but, rather, there are two significant principal components for this signature in this dataset. This is confirmed by investigation of the explained variance for each principal component. The explained variance for the first five PCA components are (1) 35.5%, (2) 22.7%, (3) 5.4%, (4) 3.5%, and (5) 2.7%. These numbers indicate that there are two significant principal components, since there is a large drop between components 2 and 3, but not between any of the later components. This is in accordance with the SCREE test for deciding the number of principal components [1]. That the Mix-29/29 signature is not optimal is further exemplified in the* transferability* plot (Figure 5(d)), where there is no indication that the loadings from the mixed signature PCA model match the reference values.

The question then arises: what does the mix signature describe: Gender-29, TvsN-100, or a mix of both of these? Exploring the correlation between the different PCA models shows that the first PCA component is the TvsN-100 signature, *r*^2^ = 0.986, and that the second PCA component is the Gender-100 signature, *r*^2^ = 0.989. This also implies that, for this mixed signature, the first component is not a mix of the two signatures but is, instead, predominately one of them.

### 3.10. Investigation of the Stability for the Mixed Signature

**Figure 6:**
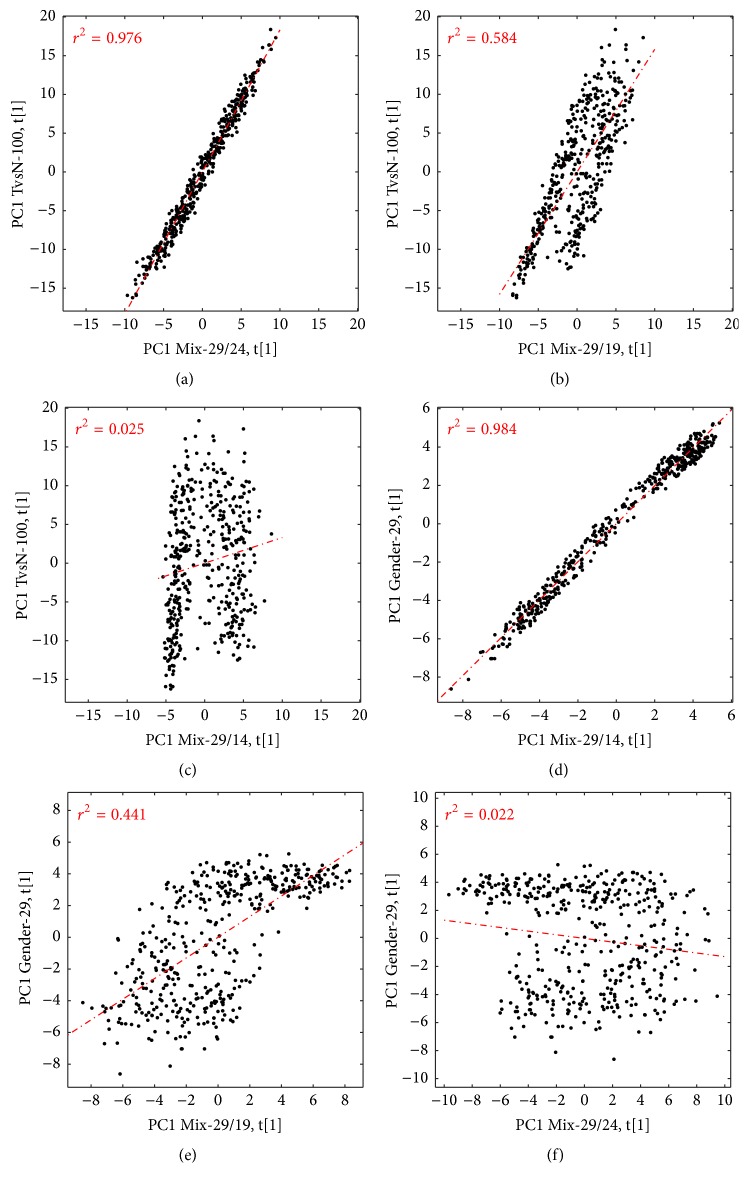
Correlation between the PCA scores from different mixed-gene signatures and the Gender-29 and TvsN-100 signature applied to the DC dataset. Even when five TvsN probesets are removed, the Mix-29/24 model is still correlated with the TvsN-100 signature (a). This correlation further decreases when more probesets are removed (b), until it is finally gone (c). The Mix-29/14 signature is, instead, correlated with the Gender-29 signature (d). The Gender-29 signature is less correlated with the Mix-29/19 signature (e) and shows no correlation with the Mix-29/24 signature (f).

When two PCA components have similar explained variance, this can cause several problems. To demonstrate this, we made small modifications to the Mix-29/29 signature by gradually removing TvsN-100 probesets, making the gender signature more prominent. The explained variance (Supplemental Fig. S6–S8) ranges between 28.5% and 35.2% for the different signatures, with the Mix-29/29 mix model having the largest explained variance. The robustness ratio is lowest for the Mix-29/19 signature PCA model, with a PC1/PC2 ratio of 1.1. This indicates that the two signatures are almost equally important for this signature. One can also see a change in the uniqueness correlation, where the Mix-29/14 signature differs from the rest: 0.087 compared to 0.331, 0.338, and 0.255. All of these findings are further confirmed in Figure 6, where the PCA scores for the different signatures are plotted against each other. The Mix-29/24 signature is describing the same biology as the original mixed signature, since the PCA scores show a high correlation (Figure 6(a)).  Figure 6(b) indicates that the Mix-29/19 signature is actually a mix of both the Gender-29 and the TvsN-100 signature, since it shows both a correlation with TvsN score and a separation between the female and male samples. If further TvsN probesets are removed, the gender signature predominates, as demonstrated in Figures 6(c) and 6(d). The Mix-29/14 signature is clearly the same as the gender signature (Figure 6(d)) and no longer related to the TvsN-100 signature (Figure 6(c)). Figures 6(e) and 6(f) further confirm that the Mix-29/24 signature is not correlated to the Gender-29 signature (f) and that only the Mix-29/19 signature is a mix of the two signatures (e).

### 3.11. Additional Validation on TCGA Dataset

To further confirm our finding, we also repeated the analysis for the Gender-29 and the TvsN-100 signature on a prostate adenocarcinoma (PRAD) dataset retrieved from TCGA. The results from these two signatures are shown in Table 1 and Supplementary Figures  9-10. It is clear from Table 1 and Supplementary Figure  9 that the Gender-29 signature does not work for the PRAD dataset. This is especially clear from the low PC1/PC2 explained variance ratio of 1.62 and that there is no correlation to the reference values, *r*^2^ = 0.025. The results are completely opposite for the TvsN-100 signature, as can be seen in Table 1 and Supplementary Figure  10. The TvsN-100 signature shows both much higher explained variance and much higher PC1/PC2 explained variance ratio than any of the random models. Furthermore, the transferability is high, with *r*^2^ = 0.904.

## 4. Conclusions

We have, in this manuscript, described the characteristics of PCA-based gene-expression signatures. Using the proposed characteristics, a signature can be validated before survival analysis or any other type of predictive modeling is done. It is important to stress the importance of validation of the signature, independent of the correlation of the signature to an outcome (e.g., survival), as was clearly shown in Venet et al. [2]. Too often, statistical significance of the signature with an outcome is used as the criteria for whether or not the signature “worked.” This is, of course, not a replacement for real biological testing that the signature is accurately predicting what it is intended to. We see this as a set of minimal requirements that any PCA-based gene signature must fulfill before moving forward with the signature in a given dataset.

Using PCA to summarize the expression of several genes has proven to be useful by others, and we also show, herein, that it is stable to random noise. However, the same properties of PCA that contribute to this usefulness can also potentially lead to misinterpretation of the signature, as shown by Venet et al. [2]. One of the most important findings presented here is that complex signatures, signatures describing multiple events, do not work well with PCA. There is always a temptation to include more genes in a signature, in order to limit the effects of outlier genes, as well as thinking that including more biologically relevant genes should result in a more stable signature. The results presented show quite the opposite, that when using PCA, a complex signature is actually less robust. The PCA will describe just one of the biological events, and which one is represented can change from dataset to dataset (Figure 6). Only if one has a perfect balance will there be a mix of the signatures (Figure 6(b)). This result is not surprising if one considers the properties of PCA. The first PCA component describes the direction in the dataset with the largest variation. The second PCA component is orthogonal to the first one and describes the second largest direction. If there are multiple biological events presented in the signature and they are not related to each other, they will thus end up in individual PCA components. The first PCA component will generally not be a combination of multiple biological events. The* robustness* plot addresses this by looking at the ratio of explained variance between PC1 and PC2. There are, of course, many ways to estimate the number of significant principal components, many reviewed in references [26, 27], but the ratio of PC1/PC2 clearly indicates if the first PCA component is describing more explained variance than the second one.

Another important finding is that it is necessary to verify that the gene signature describes the same biology, when applied to new datasets, as it was derived for: in other words, that is it* transferable*. In many cases, the signature is derived from cell line experiments where something has been perturbed. The genes that show a significant change between the control and perturbed cell lines are then used as a signature. One important note is that, in a cell line system, there is much less variation compared to what is seen in, for example, tumor data. In the cell line experiment, everything is controlled and there is only one cell type. On the other hand, in tumor data, there will be much more variation from different cell types, intracellular signaling, and immune response, to name a few. A gene selected from the cell line experiment may have a very distinct expression pattern, but, in a tumor, the expression may be dependent on many more biological effects not present in the cell line experiment. This was also shown in the paper by Venet et al. [2], where many of the tested signatures were correlated to cell proliferation, even if they were derived for describing other types of biology. It has been shown, especially in breast tumors, that one of the strongest signals in tumor expression data is proliferation [2, 28, 29]. It is then easy to see that if a signature describes a weak signal, or is not distinctive, the PCA model will detect the proliferation effect in that signature as the first PCA component. This can be easily spotted by comparing the expression pattern in the cell line experiments with those seen in the tumor data, hence the need for the transferability plot. If there is no correlation seen in this plot, it indicates that when applied to a dataset, it does not work as expected. This could be due to several reasons, such as that it may be a good signature, but it was applied to a nonrelevant dataset, for example, the gender signature on the Breast dataset. Using the reference values also solves the problem with sign-flipping between PCA models.

The* uniqueness* plot is another indicator of if the PCA-based gene signature describes the general direction in the dataset. This is an issue if the same general direction is also predictive of outcome, as it was in the case presented by Venet et al. [2]. They showed that any random signature was as good as most of the real signatures in survival analysis. This does not mean that the signature is not working, but one cannot claim that the biology it represents is predictive, since any random signature would also be as predictive. This measure of uniqueness can be extended to include a set of validated and distinct gene-sets describing major effects seen in tumor biology, such as proliferation, epithelial-to-mesenchymal transition (EMT), or immune response. With such an analysis, it would be possible to see how the new gene signature compares to already available and validated signatures.

Lastly, the* coherence* plot addresses the fact that the genes within a gene signature should be expressed in a coherent way when applied to a dataset. In the derivation of a gene signature, this is commonly one of the criteria used to select the genes for the signature. Examples include selected genes that are correlated to EC50 values from the NCI-60 data or genes that are coherently expressed across several different conditions. If this is not true, when applied to another dataset, this is manifested by a low and similar explained variance compared to the randomized gene signature PCA models. A low value of the explained variance can also indicate that there are many nonrelevant genes in the signature.

We also feel that the use of a large set of randomized gene signatures enhances the results. Many of the PCA statistics used here, like explained variance, are dependent on many factors, including sample size, number of genes used, and the general behavior of the dataset. By comparing the results from the true gene signature PCA model to the results from the randomized gene signature PCA models, this problem is minimized. It also directly addressed the question if the true gene signature is better than any random signature. It is also true that a random model can be as good as the true signature, seen in Figure 3 in this article and also in the study be Venet et al. [2].

The proposed methodologies do not remove the need for the usual best practices when it comes to using PCA to analyze data. Use score plots to find outliers, groupings, and other trends in the data that are not from biological variation, such as RNA quality and other types of batch effects. Use loading plots to see if all probesets are important for the first component, or if only some are important for later components. Furthermore, validation of survival analysis *p* values using randomized random models is also recommended, as recently pointed out by Brulard and Chibon [30] or Venet et al. [2].

## Supplementary Material

Additional validation plots for all signatures and datasets presented in table 1.

## Figures and Tables

**Table 1:** The measures of coherence, robustness, uniqueness, and transferability applied to all datasets for all signatures used.

Signature	Dataset	Coherence		Robustness		Uniqueness		Transferability
		Signature PC1 (%)	Random mean PC1 (%)	Signature PC1/PC2	Random mean PC1/PC2	Signature correlation	Random mean correlation	*r* ^2^
Gender-29	DC	45.3	10.9	4.57	1.29	0.048	0.585	0.830
Gender-29	Breast	11.8	12.3	1.32	1.31	0.804	0.606	0.000
TvsN-100	Breast	46.7	9.3	5.28	1.29	0.036	0.815	0.919
TvsN-100	DC	58.5	8.0	9.70	1.27	0.313	0.774	0.975
TvsN-50/50	DC	33.3	8.0	7.09	1.27	0.345	0.779	0.932
TvsN-25/75	DC	18.8	8.0	3.02	1.27	0.385	0.777	0.866
TvsN-10/90	DC	9.4	8.0	1.40	1.27	0.555	0.779	0.634
Mix-29/29	DC	35.2	8.9	1.55	1.29	0.331	0.697	0.008
Mix-29/24	DC	32.1	9.1	1.30	1.28	0.338	0.676	0.003
Mix-29/19	DC	28.5	9.3	1.07	1.28	0.255	0.660	0.225
Mix-29/14	DC	30.7	9.6	1.22	1.29	0.087	0.644	0.598
Gender-29	PRAD	30.8	20.4	1.62	1.68	0.825	0.777	0.025
TvsN-100	PRAD	51.7	16.9	5.72	1.69	0.117	0.949	0.904

## Abbreviations

PCA: Principal Component Analysis
DC: Director's Challenge
TvsN: Tumor versus normal
PC1: First principal component
PC2: Second principal component
PRAD: Prostate adenocarcinoma.
