# Supplementary material for: Characteristics and Validation Techniques for PCA-Based Gene-Expression Signatures
Source: Int J Genomics. 2017 Feb 6;2017:2354564. doi: 10.1155/2017/2354564 (PMC5317117; doi:10.1155/2017/2354564)
Supplement: Supplementary file 1 — Additional validation plots for all signatures and datasets presented in table 1. [file 2354564.f1.pdf]

Supplementary Figures

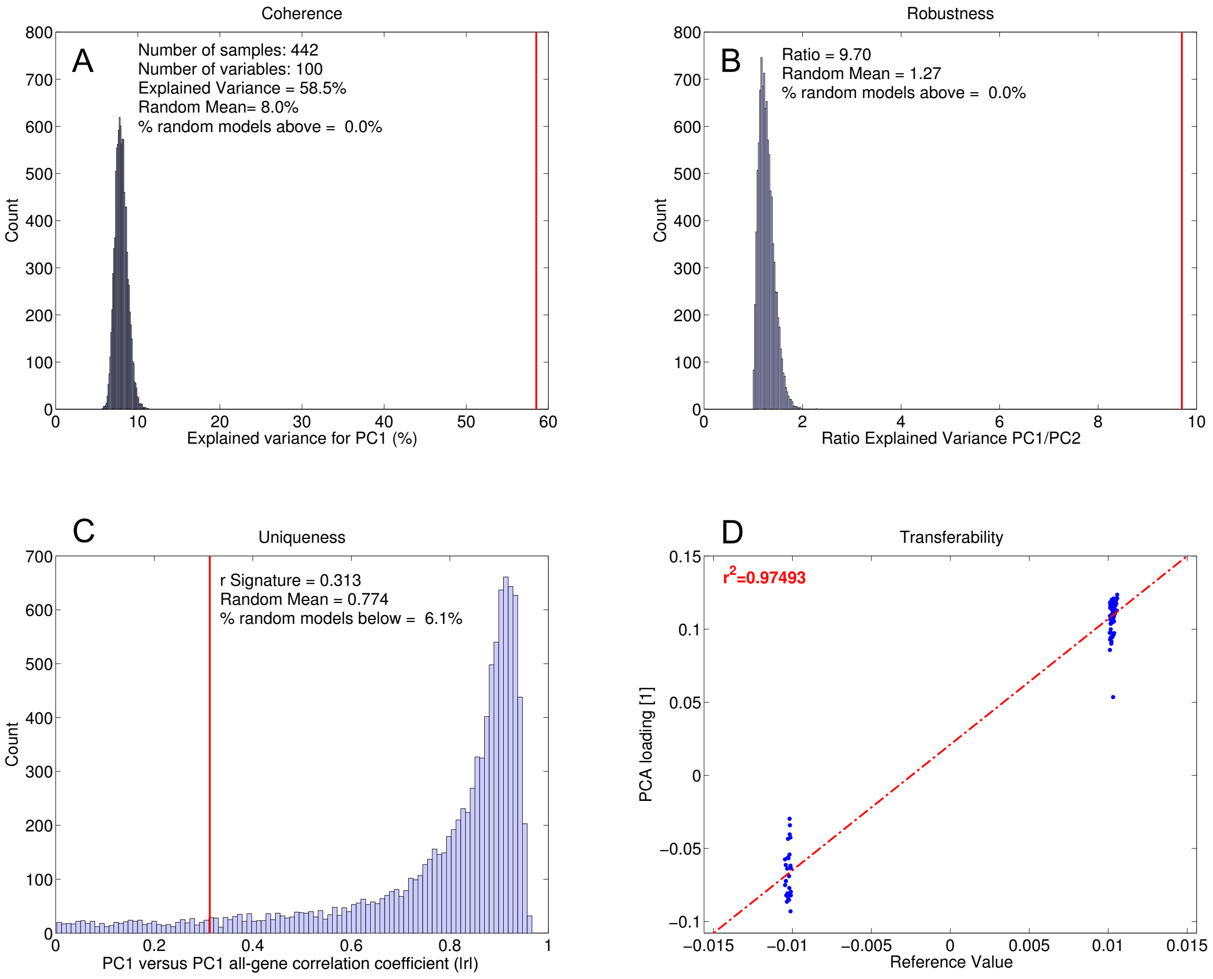

Figure S1. Validation plot for TvsN-100 signature applied to DC dataset. The TvsN-100 gene signature also performs well in the DC dataset. The probesets for the TvsN-100 signature are coherent (A), and the PCA model is robust (B). The signature is also unique (C), and describes the same biology as in the dataset it was trained upon (D).

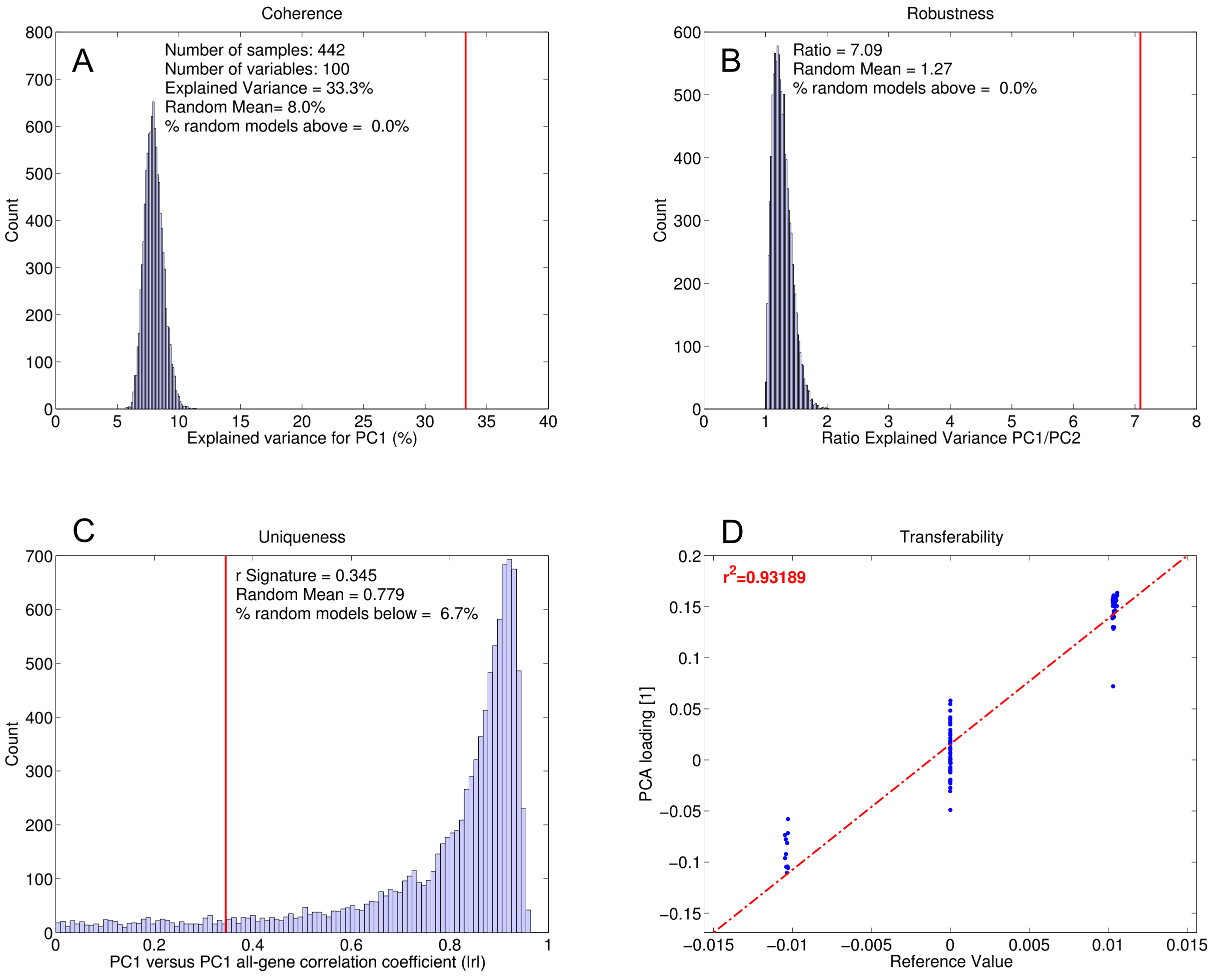

Figure S2. Validation plot for TvsN-50/50 signature applied to DC dataset. With 50% non-informative probesets, the TvsN-50/50 signature is still similar to the TvsN-100 signature. The coherence (A), robustness (B), uniqueness (C), and transferability metrics (D) all indicate that it is still a valid signature. The PCA loadings for the added non-informative probesets are all around zero (D).

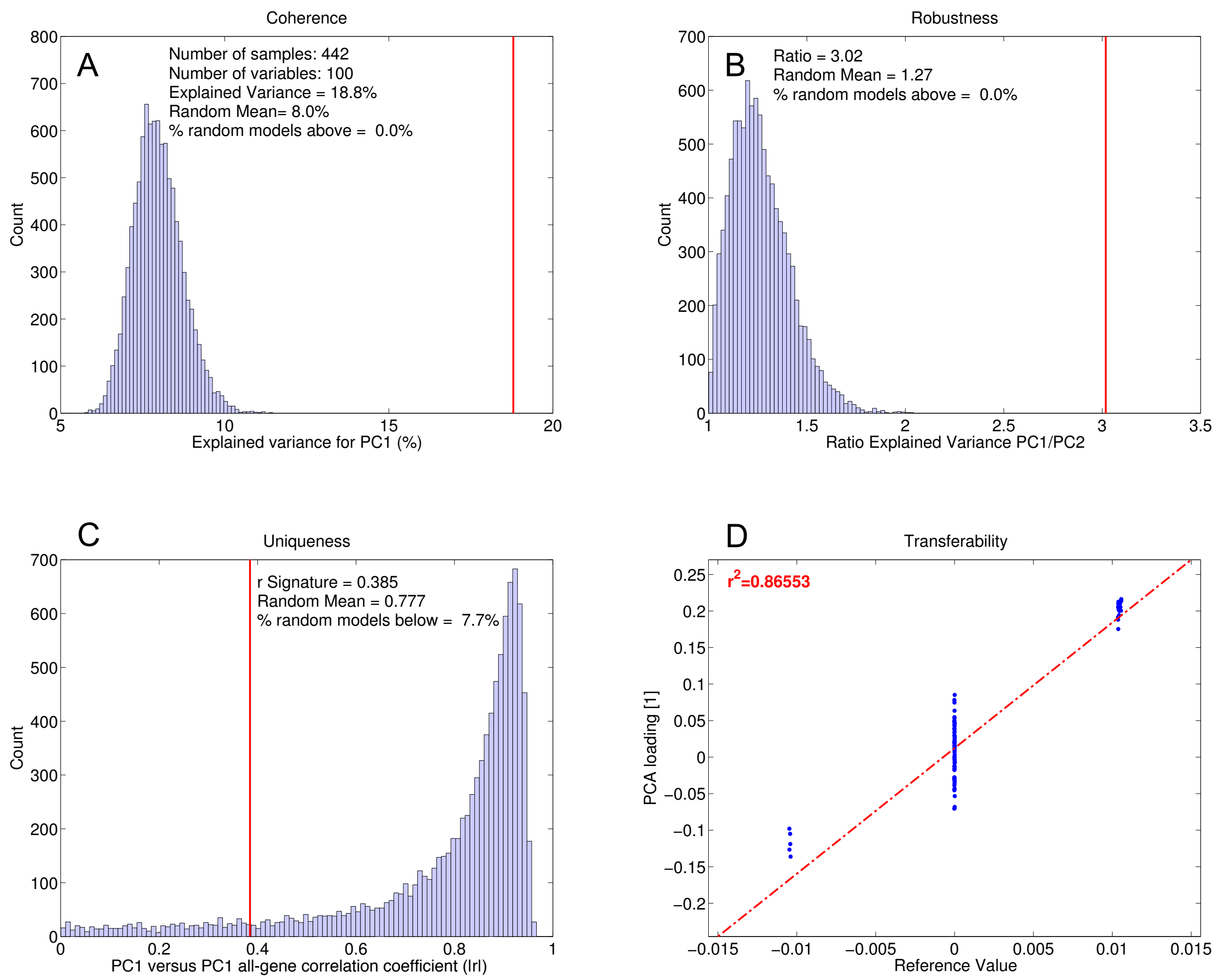

Figure S3. Validation plot for TvsN-25/75 signature applied to DC dataset. With 75% non-informative probesets, the TvsN-25/75 signature is still different from random signatures. The explained variance is still above any random model (A), even if it is no longer as high as the TvsN-100 signature. The same is also true for the robustness (B). The correlation to the general direction of the dataset is still similar to that of the TvsN-100 model (C). The PCA loadings for the non-informative probesets are still centered around zero (D).

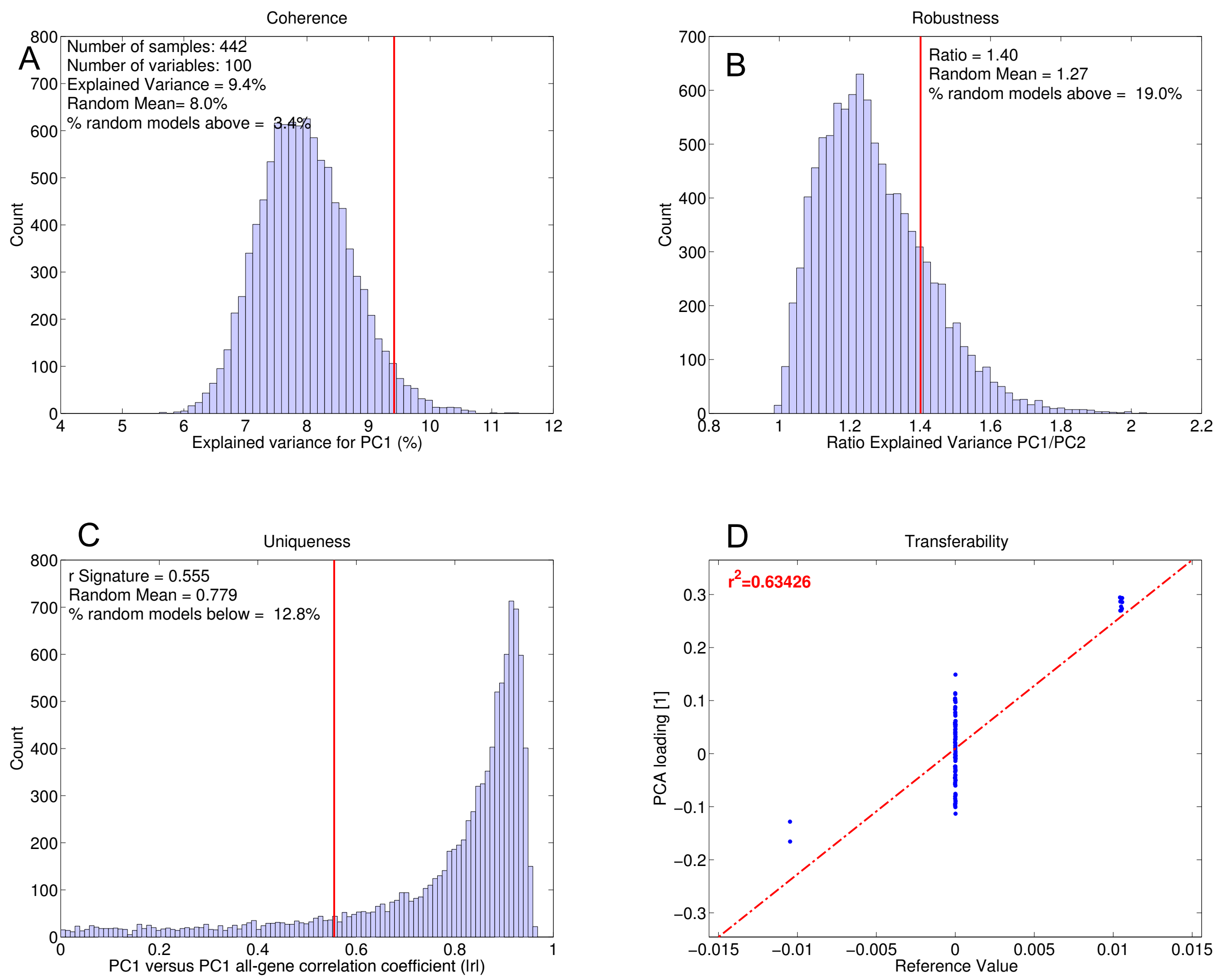

Figure S4. Validation plot for TvsN-10/90 signature applied to DC dataset. With 90% non-informative probesets, the TvsN-10/90 signature looks more like a random model. The explained variance for the TvsN-10/90 signature is lower, and 3.4% of the random model has a higher value (A). The robustness has also dropped to 1.4, with 19% of the random models having a higher value (B). The correlation to the general direction of the dataset has also gone up (C).

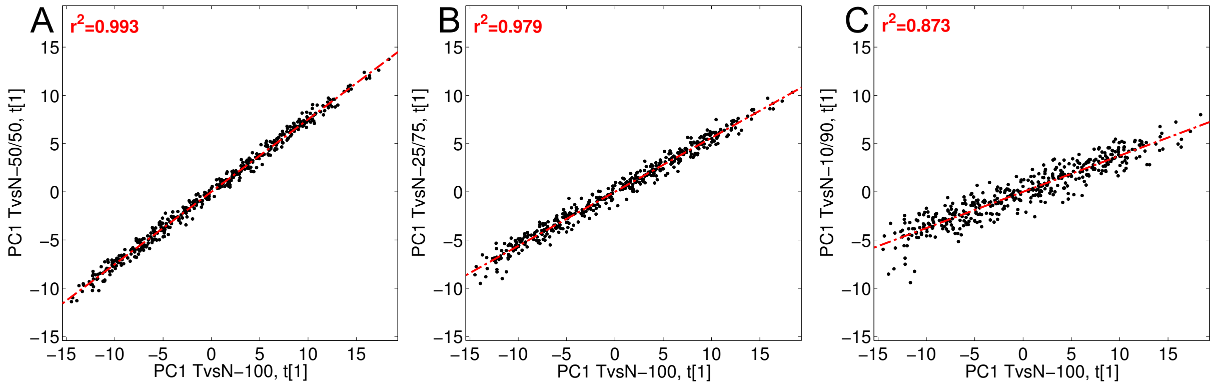

Figure S5. Correlation between the PCA scores from different TvsN gene signatures applied to the DC dataset. The PCA model is robust to the addition of non-informative probesets to the gene signature. With half of the probesets being non-informative, the scores from the TvsN-50/50 PCA model are highly correlated with the TvsN-100 signature (A). This high correlation is also seen for the TvsN-25/75 gene signature (B). Not until 90% of the probesets are non-informative does the r^2 drop below 0.9 (C).

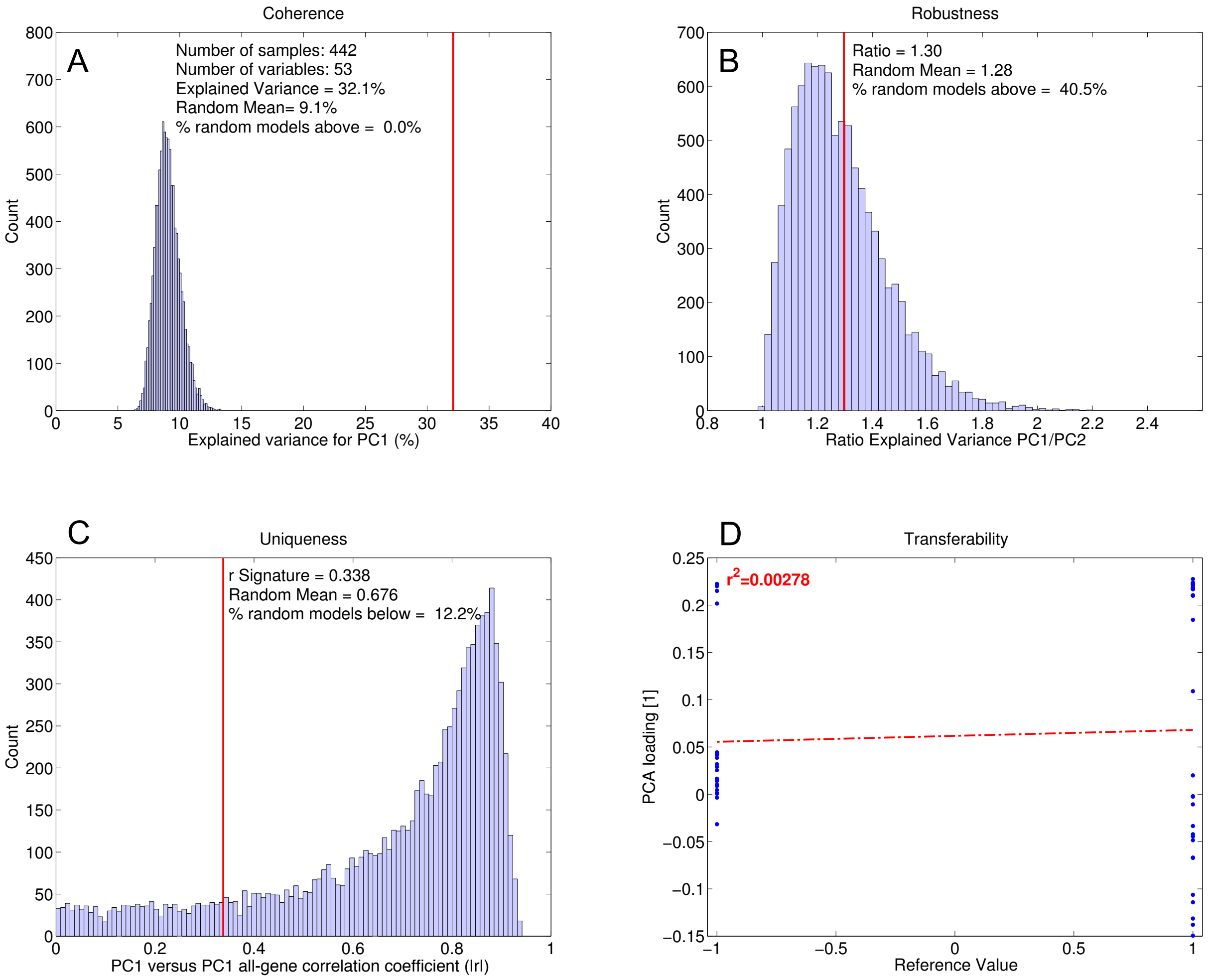

Figure S6. Validation plot for Mix-29/24 signature applied to DC dataset.

The Mix-29/24 signature is similar to the Mix-29/29 signature in its validation plots. The coherence is slightly lower (A), compared to the Mix-29/29 signature. The robustness plot is also lower, and indicates that this PCA model is not stable. The uniqueness is nearly the same (C). The transferability plot clearly shows that the model does not capture the biology described by the original signatures (D).

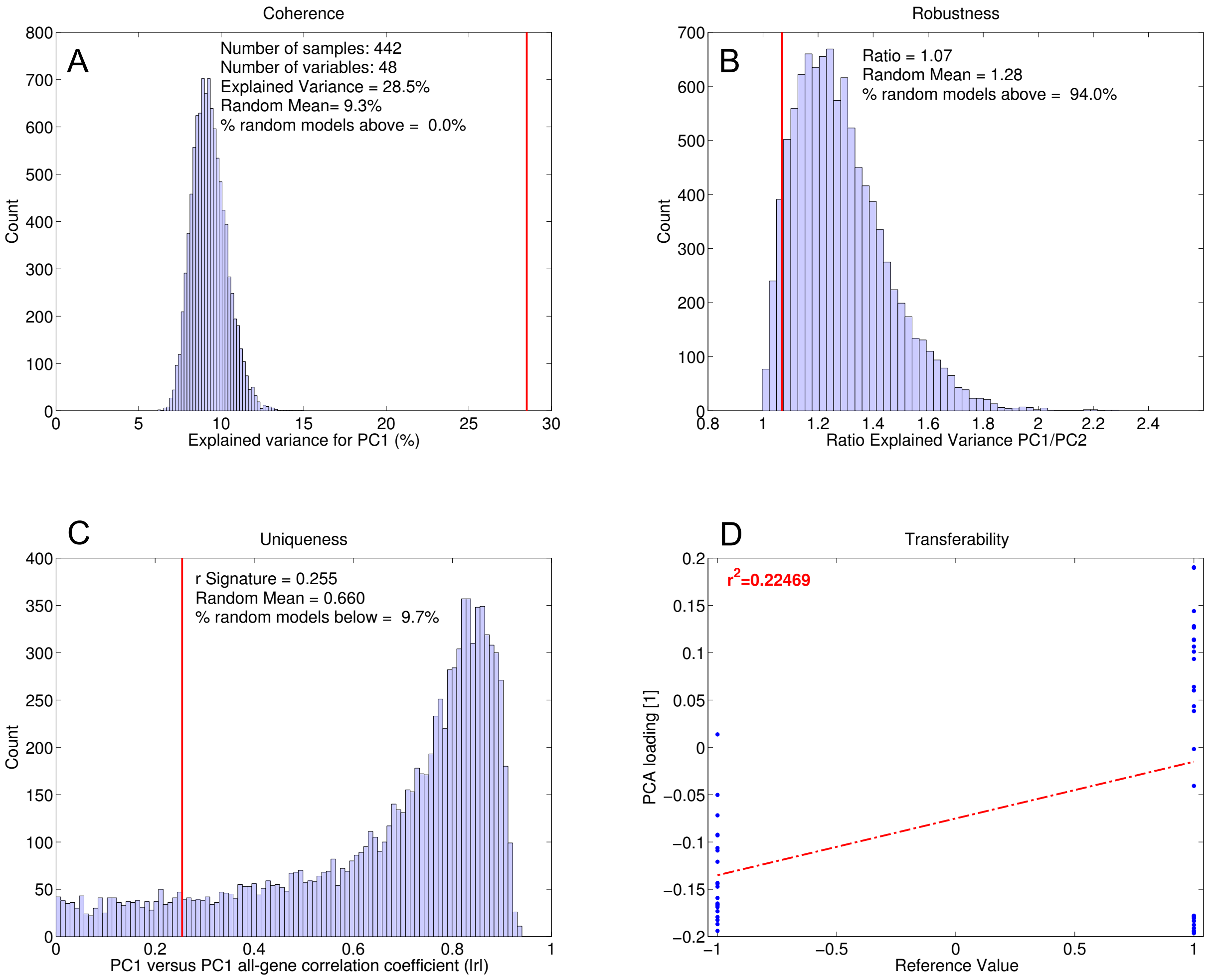

Figure S7. Validation plot for Mix-29/19 signature applied to DC dataset.

The Mix-29/19 signature is the least similar of the modified Mix signatures when compared to the Mix-29/29 signature. The coherence is somewhat lower (A). The robustness plot shows that this is an unstable PCA model, since PC1 and PC2 have similar explained variance, with a ratio of 1.07. This means that which is PC1 and PC2 can flip between datasets. The uniqueness is similar (C). The transferability plot shows that the model does not describe the biology present in the original gene signatures (D).

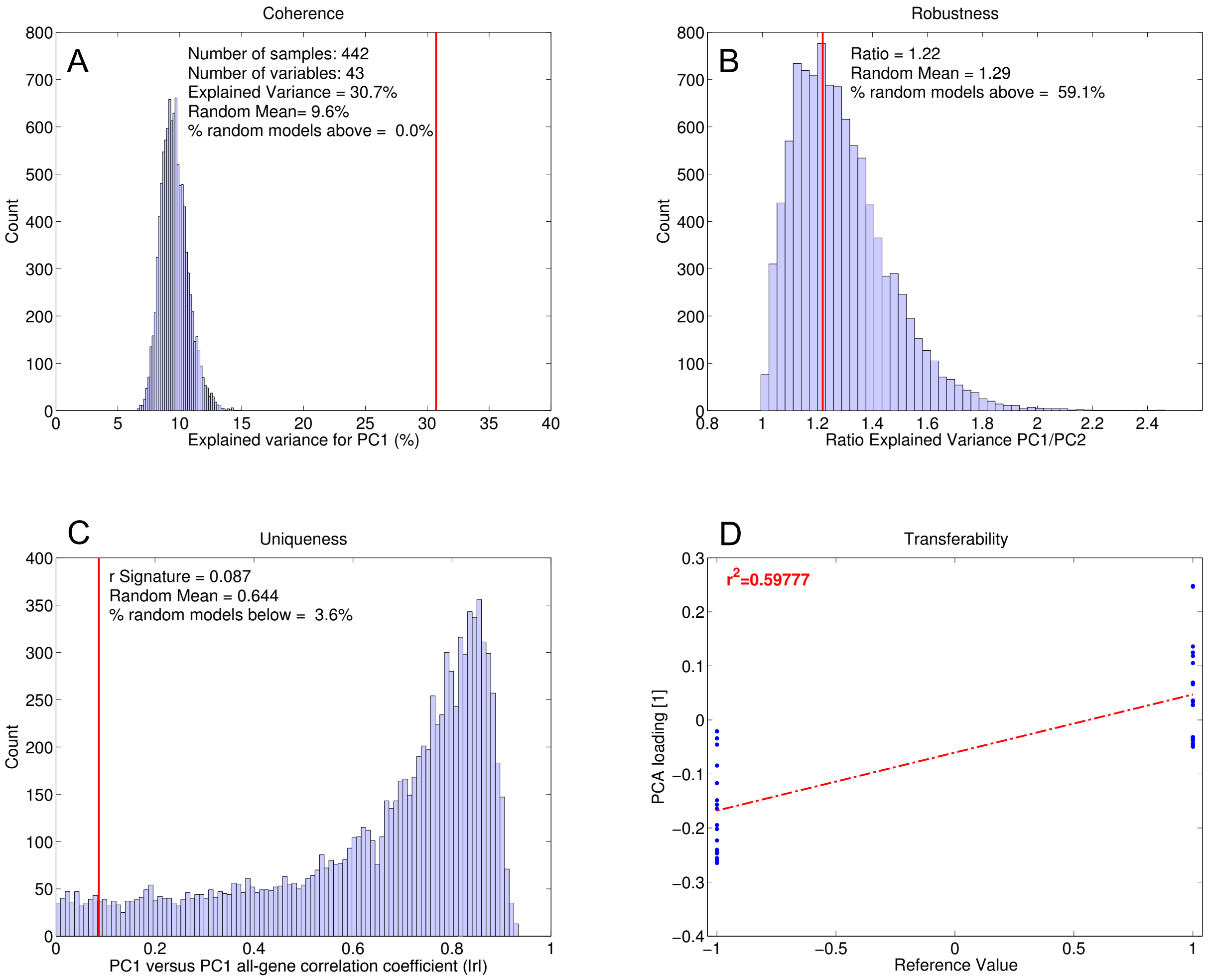

Figure S8. Validation plot for Mix-29/14 signature applied to DC dataset.

The Mix-29/14 signature validation plot is similar to that of the Mix-29/29 signature, even though it now describes a completely different direction, as seen in Figure 7D. The coherence is only slightly worse (A), and the uniqueness is noticeably improved over the Mix-29/29 signature (C). However, the robustness (B) indicates an unstable signature. Interestingly, the transferability plot exhibits some correlation, which was entirely absent in the Mix-29/29 signature (D).

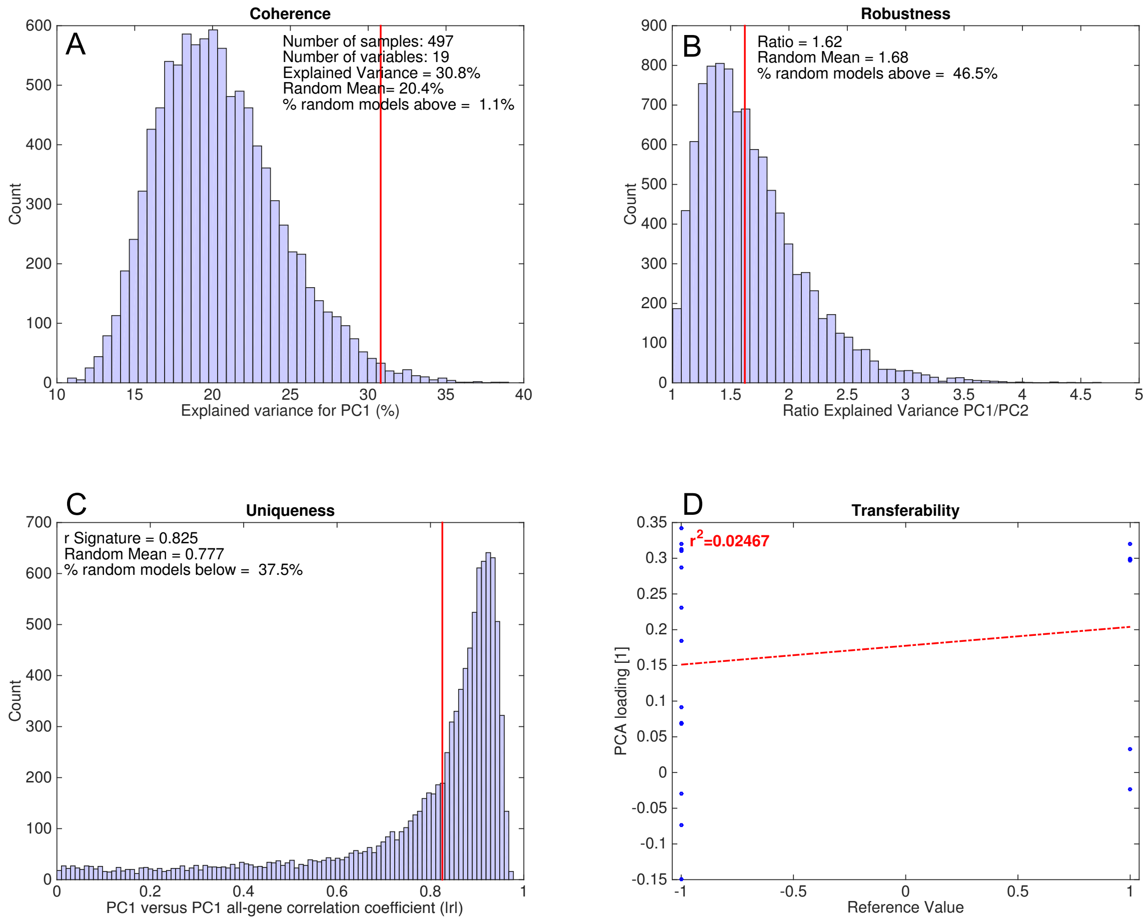

Figure S9. Validation plot for Gender-29 signature applied to TCGA Prostate dataset. The Gender-29 gene signature fails in the TCGA Prostate dataset. The genes for the Geneder-29 signature are not coherent (A), and the PCA model is not robust (B). Furthermore, the signature is not unique (C), and does not describe the same biology as in the dataset it was trained upon (D).

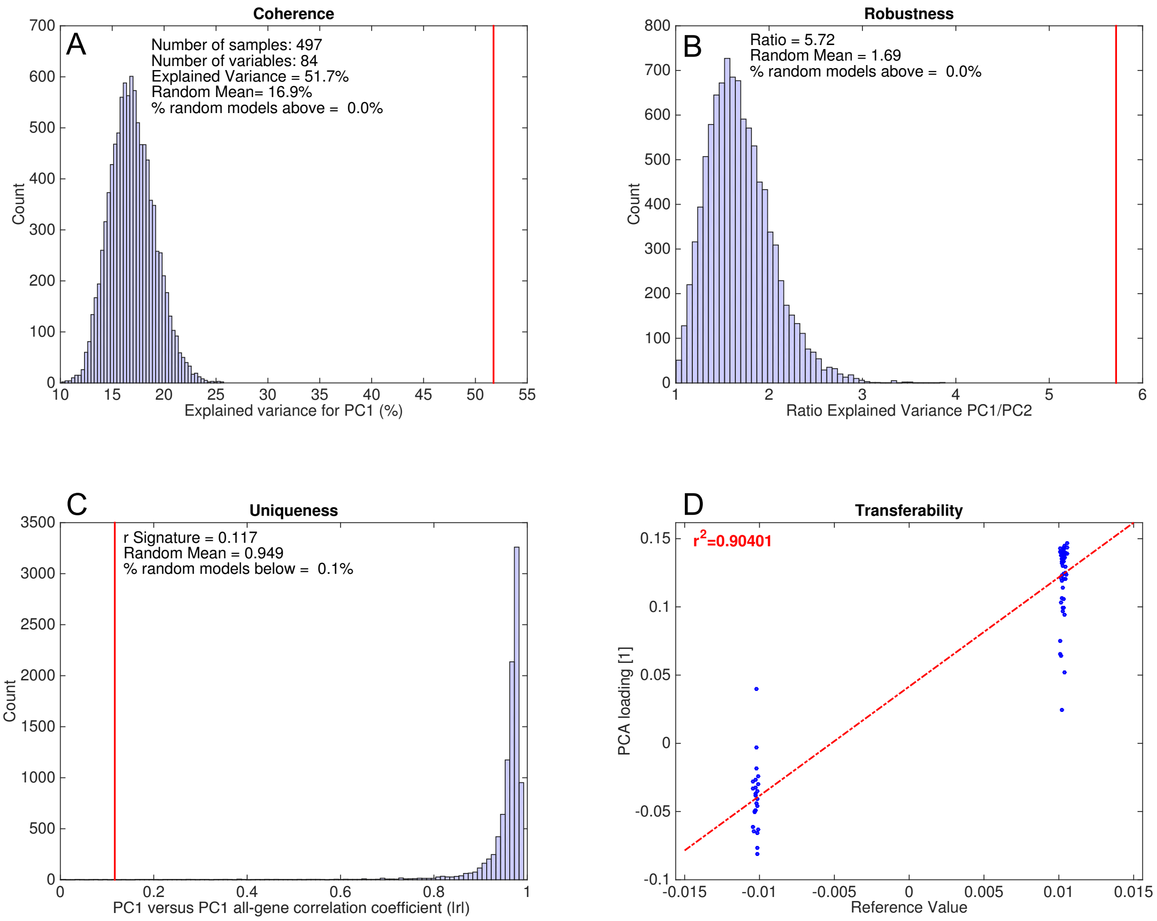

Figure S10. Validation plot for TvsN-100 signature applied to TCGA Prostate dataset. The TvsN-100 gene signature also performs well in the TCGA Prostate dataset. The genes for the TvsN-100 signature are coherent (A), and the PCA model is robust (B). The signature is also unique (C), and describes the same biology as in the dataset it was trained upon (D).
